# Modeling of the Influence of Operational Parameters on Tire Lateral Dynamics

**DOI:** 10.3390/s22176380

**Published:** 2022-08-24

**Authors:** Manuel Alcázar Vargas, Javier Pérez Fernández, Ignacio Sánchez Andrades, Juan A. Cabrera Carrillo, Juan J. Castillo Aguilar

**Affiliations:** Department of Mechanical Engineering, University of Malaga, 29071 Malaga, Spain

**Keywords:** vehicle dynamics, tire model, thermal tire model, transient tire model, relaxation length

## Abstract

Tires play a critical role in vehicle safety. Proper modeling of tire–road interaction is essential for optimal performance of active safety systems. This work studies the influence of temperature, longitudinal vehicle speed, steering frequency, vertical load, and inflation pressure on lateral tire dynamics. To this end, a tire test bench that allows the accurate control of these parameters and the measurement of the variables of interest was used. The obtained results made it possible to propose a simple model that allowed the determination of relaxation length as a function of tire vertical load and vehicle linear speed, and the determination of a representative tread temperature. Additionally, a model has been proposed to determine the lateral friction coefficient from the aforementioned temperature. Finally, results also showed that some variables had little influence on the parameters that characterize lateral dynamics.

## 1. Introduction

Vehicle safety is a subject of significant interest in today’s society. More and more, efforts are focused on active vehicle safety in the automotive industry. Within active safety, tires play a crucial role. In 2012, the NHTSA reported that tire-related defects were linked to more than 200,000 accidents per year [1]. In 2007, Tire Pressure Monitoring Systems (TPMS) became mandatory in the USA. These systems have also been mandatory in the EU since 2012. These requirements aim to ensure that tires run in optimum operating conditions to improve vehicle safety on one hand and reduce energy consumption and tire wear on the other [2]. 

In the EU, anti-lock brake systems (ABS) have been mandatory for cars and motorcycles since 2002 and 2016, respectively. In addition, electronic stability programs (ESP) have been mandatory for cars since 2014. All these factors justify the need to research and develop improvements in active security systems that consider tire–road interaction. These systems are arguably the most complex ones to determine and control. Hence, a better understanding of tire dynamics leads to an improvement in active safety systems. Therefore, the objective of this work is to analyze the effect of various parameters on cornering stiffness and tire relaxation dynamics, as well as in the maximum friction coefficient.

The modeling of tire–road interaction has been a subject of great interest over recent decades. These models are usually classified into two non-hermetic categories: empirical and physical models. The state-of-the-art model is the well-known Pacejka’s magic formula (MF) [3], which belongs to the first category. This model, valid for quasi-stationary simulations, fits the obtained experimental results reliably on and off the bench. This model has evolved over the years, from the first formulation in 1987 [4] to the current version that belongs to Siemens SimCenter Tire. Since this latest version, it has been included in the Simcenter MF-Tyre/MF-SWIFT package. One of the problems of this current version is that it does not consider any thermal parameters of the road or tire (tread, carcass, air, etc.) and linear speed of the vehicle is marginally considered in the model. Hence, several authors have modified this model to include these parameters.

Among these modifications, the following can be highlighted. Mizuno [5] proposed a linear dependence of parameter D with temperature. Sorniotti [6] proposed a linear dependence of the maximum lateral and longitudinal friction coefficient on tread temperature and a linear dependence of longitudinal and transverse stiffness on carcass temperature.

Sakhnevych et al. [7,8] proposed a modification of Pacejka’s magic formula (MF-evo) that incorporates the influence of temperature and pressure, modifying various coefficients of the MF by adding polynomial terms. The Kelly and Sharp model [9] uses Gaussian functions, modeling the friction coefficient as a function of both temperature and longitudinal velocity. The model of Harsh and Shyrokau [10] slightly modified the Kelly and Sharp model to estimate heat generation due to friction and modified the D and K parameters of the MF. D and K are affected by a quadratic factor for pure longitudinal behavior. D is also affected by a quadratic factor for pure lateral behavior, while K is affected by a more complex expression. Ozerem and Morrey [11] employ the brush model, along with Kelly and Sharp’s contact force formulation for a FSAE vehicle. Calabrese et al. [12,13] modified MF parameters λ_μ_ and λ_k_ to consider the influence of temperature in the maximum friction coefficient and cornering stiffness. Cabrera et al. [14] proposed a decreasing exponential dependence of the maximum friction coefficient on linear velocity of the vehicle.

Regarding physical models, the brush model [3] discretizes the contact patch into a series of bristles aiming to understand what happens during tire–road interaction. Romano et al. have employed a modified brush model to investigate transient effects of the tire force generation, including non-linearities [15,16,17]. Semi-physical models have also been used to study the influence of vertical load excitation frequency on the generation of contact forces [18]. Recently, with the development of finite element methods, many simulations have been carried out to study mechanical carcass deformation, pressure distribution in the contact footprint, and the generation of tangential forces [19,20]. The main problem with these methods is their computational cost, which makes it unfeasible to use them for real-time applications. In addition, a deep knowledge of tire construction is required, including: the compounds used, fiber arrangement, amount of wear, etc. Thus, FEM simulations have been relegated to the following applications: tire design by manufacturers, the study of high-frequency behavior (vibration modes) of tires, improving the understanding of mechanical/elastic behavior of tires, and, finally, allowing the generation of simplified finite element models for use in real-time applications: simulation and videogames, mainly. 

Based on experimental and FEM results, several software packages analyze tire dynamics in the following ways. The SETA tire model, used in the Project Cars 2 driving simulator, performs three simultaneous simulations: tire deformation, generation of contact forces in the tire tread and contact patch, and heat transfer and generation model [21]. Michelin’s TaMeTirE model [22,23,24] also performs three simulations, virtually identical to the SETA model. For the generation of contact forces, it distinguishes two zones: sliding and adherent. Cosin’s FTire model also performs simultaneous simulations: mechanical, thermal, wear, air volume vibration, and the flexible visco-plastic rim one [25,26]. In the iRacing simulator, the tire model currently used is the New Tire Model V7 (NTM V7). It performs the same three simulations simultaneously: carcass deformation, thread force generation, and thermal models [27].

The first methods described, the empirical ones, have the advantage that it is only under certain conditions it is necessary to measure contact forces and to access these data, either in the form of Look-Up-Tables or mathematical expressions. On the other hand, apart from the tested conditions, that is, apart from the conditions of loading, temperatures, slip, velocities, and rolling surface, the results must be extrapolated and their accuracy is not guaranteed.

Alternatively, physical models have several advantages that make them considerably more appropriate according to some authors. On one hand, the parameters that characterize tires are physical properties: stiffnesses in the three directions of space, damping coefficients, specific heats, etc. This means that modeling a new tire should theoretically be straightforward once a tire has been modeled. In addition, it is not necessary to test tires in all conditions and then interpolate them within that tested range. Nevertheless, it could be extrapolated as it is based on a physical model. The main drawback is that it is necessary to know how the tire carcass will deform according to applied loads (mechanical model) and, more importantly, to understand how the thread compound will generate frictional forces according to operating conditions. This latter point is the one that presents the most uncertainty, as indicated by all authors. The main reason is the highly non-linear viscoelastic behavior of vulcanized polymers [28].

The generation of friction forces between the tire and road is due mainly to molecular adhesion and indentation [9,29]. The first occurs on a microscopic scale due to Van der Waals bondings between the tire and road. The road must be clean and dry for this to take place. The second one, which occurs on a macroscopic scale, depends on the indentation of road roughness on the tread. Viscoelasticity of the compound means that, as roughness penetrates rubber, it is necessary to apply more energy than it releases when it returns to its original shape. This difference causes a resulting force that opposes sliding. Therefore, viscoelasticity generates friction between the tire and road. Hence, the importance of viscoelastic behavior of the compound. What happens is that this viscoelastic behavior is strongly dependent on a multitude of factors, which explains the enormous complexity of determining contact forces. Essentially, this friction depends on the temperature of tires with respect to the vitrification temperature of the compound, the frequency of excitation of these irregularities and the chemical composition of the compound. When excited at low frequencies, the tire behavior is essentially elastic, and the viscous component is of little importance. At the other extreme, the tire is dramatically stiffer at very high excitation frequencies, behaving more like glass (glassy state). In an intermediate frequency range, the viscoelastic component predominates. It is in this frequency range where it is essential to work. Something similar happens with temperature. Below the vitrification temperature (the temperature at which it changes from one kind of behavior to the other), the composite is very rigid and brittle. The behavior is purely elastic above the vitrification temperature, with a much lower stiffness than when it is colder.

The vitrification temperature in winter tires is considerably lower than the one in the case of summer tires, which, in turn, is lower than the one in competition tires (slicks). William, Landel, and Ferry [30] determined an expression to relate these variables (WLF equation) since this vitrification temperature also depends on the excitation frequency. This relationship, which has been known for decades and is applied to polymers, has been adapted to the field of tire dynamics, replacing working temperature with respect to vitrification temperature with a working temperature with respect to a reference temperature, as well as the excitation frequency by sliding velocity or longitudinal velocity. This is the case of the TaMe-TirE model or the Sharp and Kelly model. Other models, such as those used in simulators, being a black box, do not specify which model of tangential force generation they use. However, they consider temperature and time (iRacing, SETA,...). The relationship between the friction coefficient and sliding velocity and the temperature of the TaMe-TirE model has a Gaussian shape, similar to the one described by WLF in their work. Additionally, the Kelly and Sharp model considers a Gaussian shape.

Another area of study that has been investigated and which has garnered significant interest in recent years is the study of transient tire dynamics. Most tire data are obtained under quasi-stationary conditions. Therefore, it is impossible to predict tire behavior apart from low-frequency maneuvers. As Lozia demonstrates [31], not including transient models in vehicle control simulations can lead to inaccurate results. It is, therefore, necessary to use more complex models to consider this specific type of maneuver, such as the operation of an ABS, an ESP, or crash-avoiding maneuvers (lane change maneuver). The first works on this subject date back to the second half of the twentieth century, including those of Segel [32,33] and Schieschke [34,35]. Pacejka’s new formulation of the model is called SWIFT, which stands for short wavelength intermediate frequency tire model. Many of the efforts for this type of transient model have focused on vertical load fluctuations [18], produced by load transfer or road irregularities. Others employ very complex models where it is necessary to solve systems of differential equations, such as in the case of the SWIFT model [3], RMOD-K [36,37], or FTire [25,26]. A comparison of these three methods can be found in [3]. Luty [38] analyzes the influence of relaxation parameters for truck tires on the results of a simulation. Other authors use first [39] or second order models [40] with lower computational costs. These first-order systems are a good compromise between the number of tests needed to characterize them and the accuracy of the model. In addition, the computational cost is meager. These first-order linear models are defined by proportionality and time constants. This time constant is usually expressed as a distance traveled and is referred to as the relaxation length. In this work, a linear first-order model is used, defined by the two parameters described above, which, as it will be seen, are strongly influenced by vertical load and linear velocity.

Two extremes can be distinguished within tire model formulations: pure lateral and pure longitudinal dynamics. Once these two cases have been studied, they can be used together in the so-called combined models. In the former, longitudinal sliding velocity is zero, and so is longitudinal force; in the latter, the opposite occurs: lateral sliding velocity is zero and, hence, lateral force is zero. In this work, we will analyze only the pure lateral model. This is because:

- Lateral dynamics are much more stable: it is much easier to control lateral slip than longitudinal slip. This allows for much better repeatability of tests.
- The generation of lateral forces generates a temperature gradient across the tread that the longitudinal force does not. This allows for studying the influence of temperature distribution along the tread.
- The tire test bench at the research group’s facilities cannot impose one speed on the wheel and another on the running belt. Only braking of the wheel is possible. This results in two outcomes. First, the tests requiring longitudinal slippage must be brief, as the brake disc heats up very quickly. Secondly, once the slip corresponding to maximum grip has been reached, the tire locks up almost instantaneously, making it very difficult to work in the non-linear zone of the tire. In addition, measuring angular velocity of the wheels in situations close to lock-up and, therefore, measuring high slip is quite complex in a vehicle not instrumented for this purpose [41].

Therefore, temperature and speed significantly influence the generation of contact forces on the road, being these forces the only ones that enable the vehicle to be driven. To the best of the authors’ knowledge, active safety systems in vehicles do not measure tire temperature. Only some TPMS monitor gas temperature inside the tire. This is why the objective of the present work is multiple:

- Designing, coding, and assembling the temperature measuring device using IR sensors to determine temperature distribution of the tire.
- Performing a number of tests to determine the influence of various parameters on the generation of contact forces.
- Analyzing and proposing a novel model to estimate the parameters of interest in the tire.

Regarding the measurement of tire temperature, it is interesting to measure it for the following reasons:

- It is the most easily measurable temperature and the most relevant to generation of contact forces.
- An accurate and inexpensive temperature distribution can be obtained using an infrared sensor, given that the emissivity of dry rubber is known and approximately constant.
- Only tire sidewall temperature is considered for estimating carcass temperature.
- The contributions of the present work are:
- Proposing a model for determining a representative temperature as a function of the temperature of the whole tire.
- Proposing a model for estimating relaxation length as a function of vertical load and linear speed of the vehicle.
- Proposing a model to determine the maximum friction coefficient as a function of the representative tire temperature.
- Ensuring a reduced computational cost to evaluate these models while maintaining a reduced number of tests needed to characterize them.

This work is structured as follows: first, a description of instruments employed, experiments performed, and mathematical methods is provided in the Materials and Methods section. This section and the Results and Discussion section are divided into two subsections: linear and non-linear experiments. In the first subsection, experiments are described. Later, results obtained in real tests are exposed and analyzed in the Results and Discussion section. The conclusions of this work are drawn in the last section, as well as future works.

## 2. Materials and Methods

**Figure 1:**
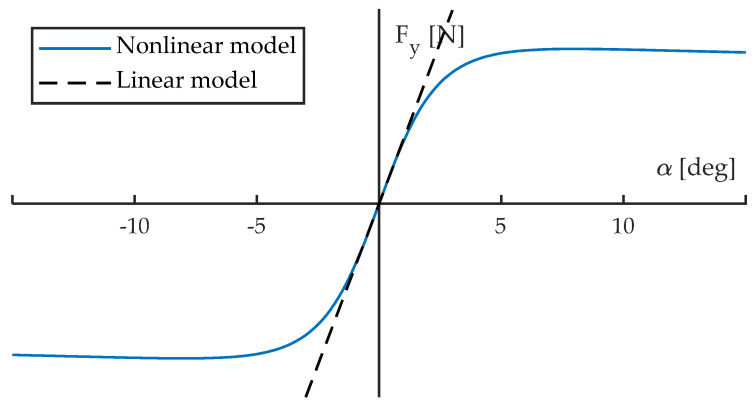
Pure lateral force against slip angle. Linear and non-linear regimes.

This section describes the tests performed. The conditions of tests, the variables that have been studied, and the instrumentation used are also explained here. It is also described how measurements were made. The tests are grouped into two main blocks: linear and non-linear (Figure 1). Linear tests are those where the slip angle, α, remains close to zero.

To characterize behavior within linear range of the tire, the following parameters are measured:

- Relaxation length for the generation of F_y_: L_Fy_;
- Cornering stiffness: C_α,Fy_.

Apart from linear range, it is also of interest to determine the maximum lateral friction coefficient, µ_y_. The objective of this work is to determine the influence of the following variables on the previous parameters:

- Vertical load: F_z_;
- Longitudinal speed: V_x_;
- Tire pressure: P;
- Tire temperature: T;
- Slip angle excitation frequency: f_SA_.

### 2.1. Linear Tests

To determine the influence of these variables on linear parameters of the tire, three groups of tests are performed, resulting in a total of 88 experiments. For all of them, unless otherwise stated, the conditions are:

- Vertical load, 4000 N;
- Longitudinal speed, 60 km/h (16.7 m/s);
- Tire pressure, 2.3 bar;
- Tire temperature, 60 °C;
- Slip angle excitation frequency, 1 Hz;
- Slip angle excitation amplitude, 2°.

In these first linear tests, a sinusoidal slip angle of amplitude and frequency, indicated above, is imposed. The amplitude of 2 degrees has been chosen to stay within the linear region. It has been decided to excite the system with a frequency of 1 Hz because it is in the middle of the excitation frequencies considered. The following first-order system is fitted (1):

(1) $$y=\frac{C}{\tau \;s+1}\;u$$

where C is the cornering stiffness, u is the slip angle, and τ is the time constant of the first order system. The output, y, is the lateral force. To determine relaxation length, L, once longitudinal velocity, V_x_, and the time constant, τ, are known, it is given by Equation (2):

(2) $$L={\tau \;V}_{x}$$

Subsequently, the Matlab System Identification Toolbox [42] is used, and the iddata test object is created. With this toolbox, these parameters can easily be determined while monitoring the error made in the modeling. 

**Figure 2:**
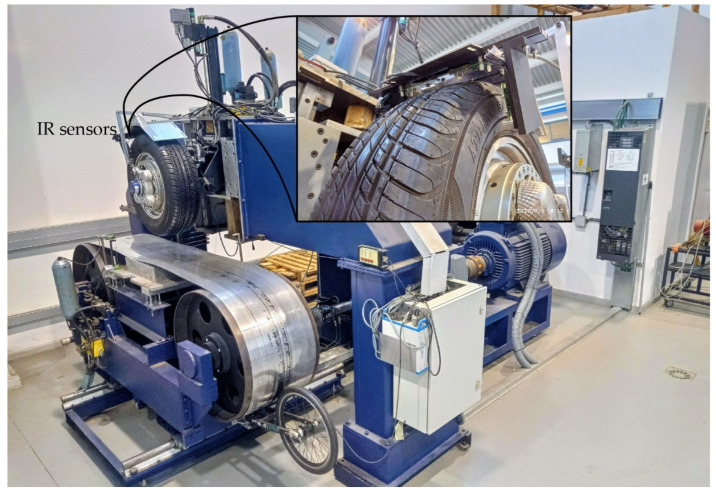
Tire testing machine property of the research group.

The tests are performed on the research group’s tire testing machine at the University of Malaga (Figure 2) [43]. Forces and torques are measured with a Kistler RoaDyn P625 instrumented rim. Control and data acquisition are performed with an sbRio 9637 and LabVIEW software. Data acquisition is performed at 5 kHz and the machine is controlled in closed loop at a frequency of 1000 Hz.

**Figure 3:**
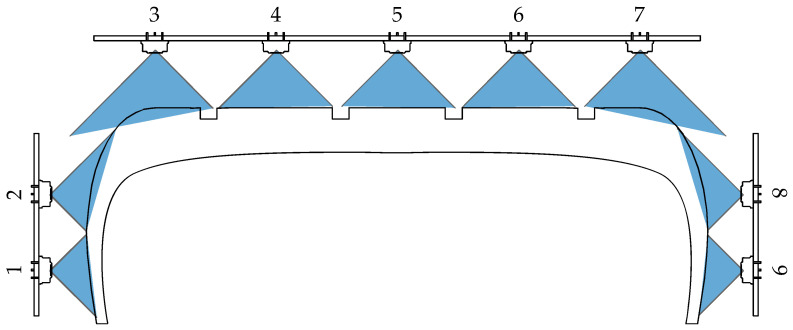
IR sensors layout.

The temperature sensors used are Melexis MLX90614. These infrared sensors provide a digital output through the I2 C protocol. Nine of these sensors are arranged on three PCBs (Figure 3). A microcontroller is used to convert transmitted messages by I2 C into a CAN message, which is much more common in the automotive industry and has no transmission distance problems. The maximum acquisition frequency of these sensors is 2 Hz. This frequency is limited by the filter of the sensor. Thus, all variables are recorded at a rate of 5 kHz, except tire temperature which is acquired at 2 Hz. Table 1 shows a fragment of the calibration certificate of the instrumented rim.

As for the tests in which the temperature is required to be homogeneous and not to vary throughout the test, the way to achieve these conditions has been as follows. A tire warmer from the manufacturer Thermal Technology with an RC31 II Series controller is mounted and the temperature is set. It is kept at this temperature for 60 min and the pressure is adjusted once the tire is hot. This way, all layers of the tire are at the same temperature. Immediately after removing the heater, the test to determine the relaxation parameters is carried out. These tests have a very short duration as only a few cycles are performed. The measured surface temperature is not shown on the graphs as it is constant. 

On the contrary, for tests to determine the maximum lateral friction coefficient as a function of tread temperature, the tire starts at ambient temperature and gradually warms up due to friction. In this case, the surface temperature is continuously heated until the maximum temperature is reached. This leads to a non-homogeneous temperature distribution, where the hardest working part of the tire heats up the most.

#### 2.1.1. First Group of Tests: Influence of F_z_ and V_x_

Different vertical loads and longitudinal velocities are imposed in the first set of tests. The loads vary from 2000 to 6000 N in 1000 N steps. The speed increases from 30 to 70 km/h in 10 km/h increments. Thus, there are five different loads and five different speeds. Therefore, 25 tests are performed.

#### 2.1.2. Second Group of Tests: Influence of Slip Angle Excitation Frequency

These tests are similar to the previous ones, with the difference that the wheel steering frequency varies from 0.1 Hz to 2.0 Hz, with increments of 0.1 Hz until reaching 1.5 Hz and 0.5 Hz after that. The reason is that a significant change in the relaxation length and stiffness is observed at around 1 Hz. This group consists of 18 tests.

#### 2.1.3. Third Group of Tests: Influence of Temperature and Pressure

The last group of tests in the linear regime is performed by modifying temperature and pressure. Tests are carried out at 30, 60, and 90 °C and 1.8, 2.3, and 2.8 bar. Vertical load is also modified since it is believed that pressure and vertical load are related, as both significantly affect the shape and distribution of the contact patch. Vertical load ranges from 2000 to 6000 N in 1000 N steps. All other parameters remain the same. Thus, three different temperatures and pressures and five vertical loads are tested, resulting in 45 tests.

### 2.2. Non-Linear Tests

Finally, to determine the influence of temperature on the maximum lateral friction coefficient, it is necessary to perform another approach. It is known that the maximum friction coefficient is outside the linear range of tire behavior [3], so it will be necessary to impose a much higher slip angle. Therefore, the following test is performed. A slip angle of −8 degrees, a longitudinal speed of 60 km/h, and a vertical load of 4000 N are set. The test starts with a cold tire that warms up during the test. After about three minutes, some parts of the tire reach 120 °C and the experiment finishes.

The test results allow determining the influence of tire temperature on lateral force generation. Two issues are addressed:

1. Taking a representative temperature value (scalar) from the reading of the nine sensors. The following Equation (3) is proposed:
  (3) $$T_{\mathrm{averaged}}=\sum_{i=1}^{n}\left(w_{i}\cdot T_{i}\right)$$
  where w_i_ is the weight corresponding to the reading of each sensor. The expression to calculate it is (4):
  (4) $$w_{i}=\frac{T_{i}-T_{\mathrm{ambient}}}{\sum_{j=1}^{n}\left(T_{j}-T_{\mathrm{ambient}}\right)}$$

It is proposed to assign a weight to the *i*-th sensor reading proportional to temperature difference between that reading and ambient temperature. This way, the tire parts at ambient temperature do not contribute to the generation of frictional force. This expression was chosen because convection is the fundamental mechanism of heat dissipation in this process (Newton’s law of cooling). The heat dissipated by this mechanism, Q, is proportional to temperature difference between of the tire surface (measured magnitude) and ambient temperature.

Thus, assuming that all the heat is generated by friction and evacuated only by convection, as shown by the experimental results by de la Rosa et al. [44], the following can be written (5):

(5) $$\mu_{y}\cdot F_{z}\cdot V_{x}\cdot \sin\alpha =Q\propto \left(T-T_{\mathrm{ambient}}\right)$$

where term $\mu_{y}\cdot F_{z}$ is lateral force $F_{y}$. It is impossible to know the pressure distribution in the contact patch during the steering process in a vehicle that is not instrumented for this purpose. Therefore, this weighting method assigns more importance to the part of the tire that is more heated since it contributes more to the generation of lateral force. Moreover, computing this operation is highly inexpensive. In this way, these weights (4) take into account the zones of the tire that generate friction. In the extreme case that the temperature of a tire zone coincides with ambient temperature (T_i_ = T_ambient_), it must be because this zone does not generate friction. Otherwise, this friction would be converted into heat which increases the temperature of that part of the tire.

2. Proposing a model to determine the grip as a function of temperature. In this case, an expression using a hyperbolic cosine is proposed, which will be described in more detail in the Results section.

## 3. Results and Discussion

The previous section described procedures for performing tests, instruments used, and data processing. This section will show the results and their interpretation. Following the previous section, they are divided into two main groups: linear and non-linear tests. A commercial summer passenger car tire is tested (Austone Athena SP-6) with an overall size of 205/65 R15.

### 3.1. Linear Tests

The results of the 88 linear tests performed are shown in Figure 4, Figure 5, Figure 6, Figure 7, Figure 8, Figure 9, Figure 10, Figure 11 and Figure 12.

#### 3.1.1. First Group of Tests: Influence of F_z_ and V_x_

**Figure 4:**
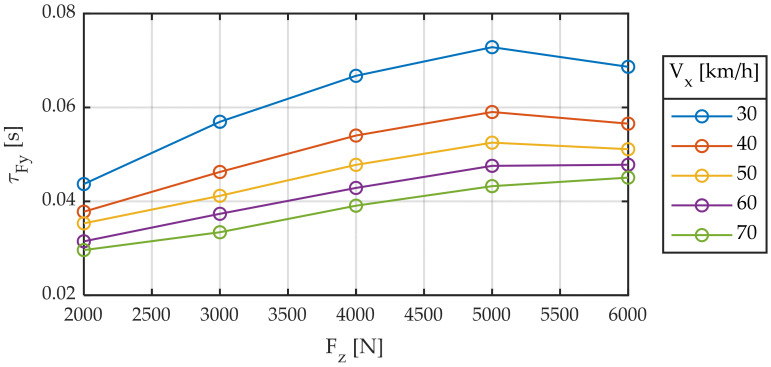
Relaxation length for different longitudinal speeds. Slip angle sweep ± 2°. Frequency 1.0 Hz.

In these first tests, the modified variables are vertical load and linear velocity. Figure 4 shows lateral relaxation length. This length is not constant but increases with vertical load and speed.

**Figure 5:**
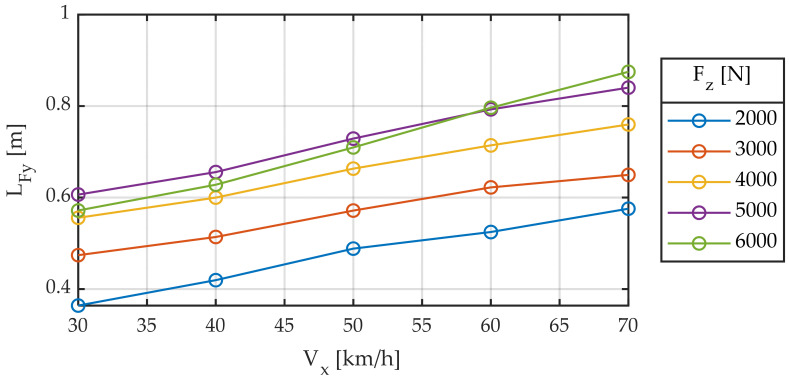
Relaxation length for different vertical loads. Slip angle sweep ± 2°. Frequency 1.0 Hz.

Figure 5 also shows the same results as in Figure 4, where the *x*-axis represents longitudinal velocity. Similar conclusions can be drawn as in the previous case, but two phenomena can also be distinguished. First, a linear dependence is observed between relaxation length and longitudinal velocity. Second, a non-linear dependence is observed between relaxation length and vertical load. 

**Figure 6:**
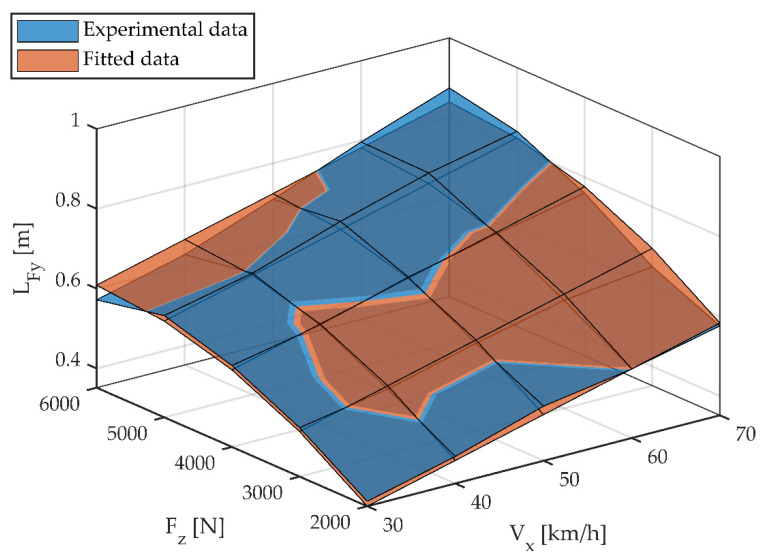
Relaxation length for different vertical loads and longitudinal speeds. Experimental data and fitted data.

Considering the results presented in the previous two figures in this work, we propose the following model (6):

(6) $$L_{\mathrm{Fy}}=c_{1}+c_{2}V_{x}+c_{3}F_{z}+c_{4}F_{z}^{2}$$

which is fitted through least squares [45], yielding the following values (Table 2):

Figure 6 shows the relaxation length as a function of vertical load and linear velocity. Experimental results and the polynomial function fitted by Equation (6) are shown. A very accurate fit can be observed.

**Figure 7:**
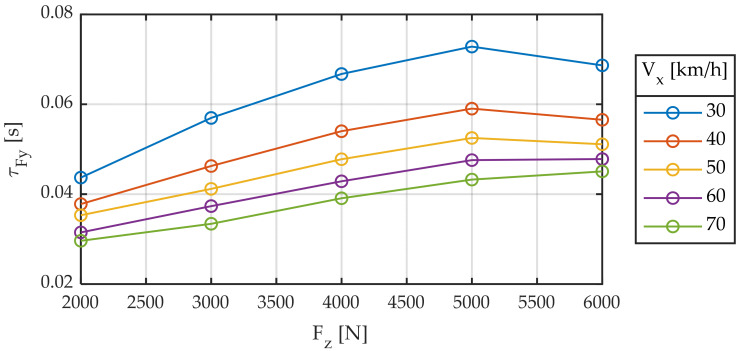
Relaxation time constant for different longitudinal speeds. Slip angle sweep ± 2°. Frequency 1.0 Hz.

**Figure 8:**
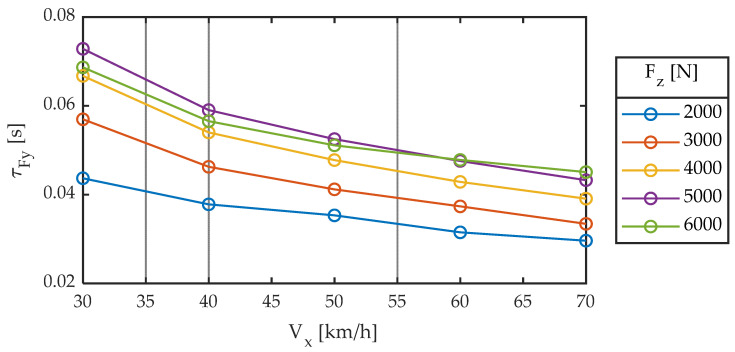
Relaxation time constant for different vertical loads. Slip angle sweep ± 2°. Frequency 1.0 Hz.

The delay in the generation of forces can be interpreted as distance (relaxation length) or time (time constant). What happens is that neither the first nor the second is constant. Figure 7 and Figure 8 are equivalent to Figure 4 and Figure 5, respectively, where the time constant has replaced relaxation length.

It is observed that the delay between the input to the system (slip angle) and the output (force) is strongly dependent on both vertical load and linear velocity and that it is not constant, whether viewed either in the form of distance traveled or in the form of time. Maximum variations in the order of 200% are observed in time (30–70 ms) and distance (0.3–0.9 m).

**Figure 9:**
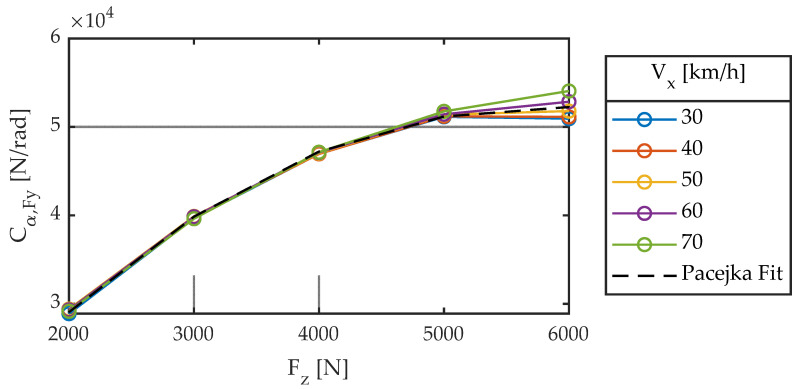
Cornering stiffness for different longitudinal speeds. Slip angle sweep ± 2°. Frequency 1.0 Hz.

**Figure 10:**
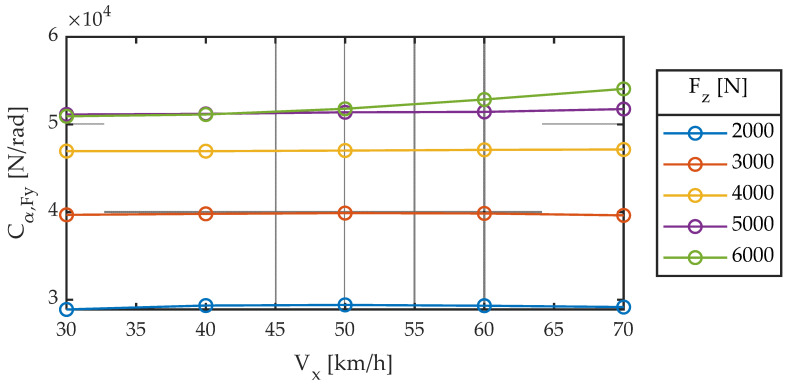
Cornering stiffness for different vertical loads. Slip angle sweep ± 2°. Frequency 1.0 Hz.

The other parameter characterizing a first-order system is the proportionality constant or gain. For the case of no camber angle (γ = 0) and without taking into account the influence of pressure (dp_i_ = 0), Pacejka [3] proposes the following Equation (7):

(7) $$C_{\alpha ,\mathrm{Fy}}=d_{1}\cdot \sin\left(d_{2}\cdot \mathrm{atan}\left(d_{3}\cdot F_{z}\right)\right)$$

whose nomenclature has been modified according to the one of the present work. This stiffness in literature does not depend on linear velocity but vertical load. Figure 9 and Figure 10 show experimental data, which agree with the Equation (7) proposed by Pacejka (dashed line). The fitted coefficients are summarized in Table 3.

#### 3.1.2. Second Group of Tests: Influence of Slip Angle Excitation Frequency

**Figure 11:**
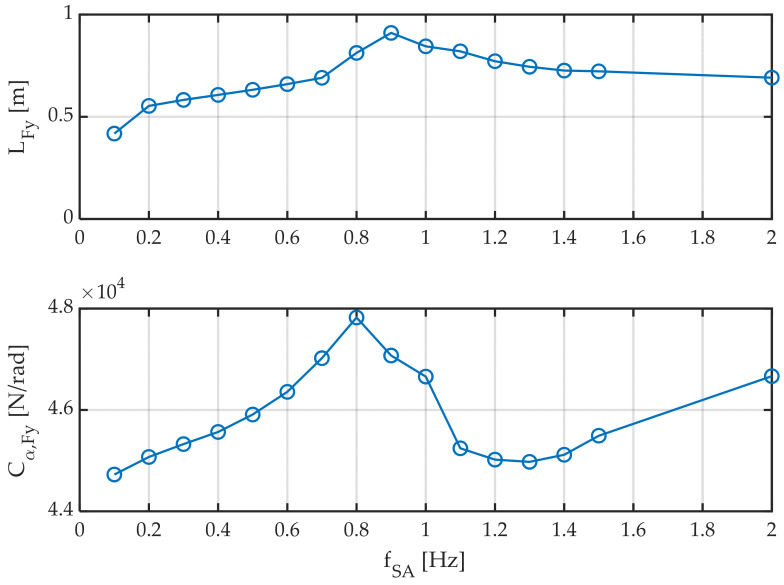
Relaxation length and cornering stiffness for different slip-angle sweep frequencies. Amplitude ± 2°. Longitudinal speed 60 km/h. Vertical load 4000 N. Tire temperature 60 °C.

The next series of tests seek to determine the influence of the slip angle excitation frequency on the two parameters studied above. The slip angle is related to the steering angle. Hence, it is interesting to determine the delay in lateral force generation depending on steering wheel speed. Figure 11 shows how relaxation length and stiffness vary as a function of excitation frequency. Several remarkable effects can be observed:

- The relaxation length increases from almost zero frequencies (<0.5 m) to a maximum at around 1 Hz, where it almost doubles its value (~1.0 m).
- The stiffness also has a maximum value of around 1 Hz, but these values vary much less in percentage terms, around 5%.

Testing was not continued at frequencies higher than 2 Hz since faster maneuvers are rare to occur. 

#### 3.1.3. Third Group of Tests: Influence of Temperature and Pressure

**Figure 12:**
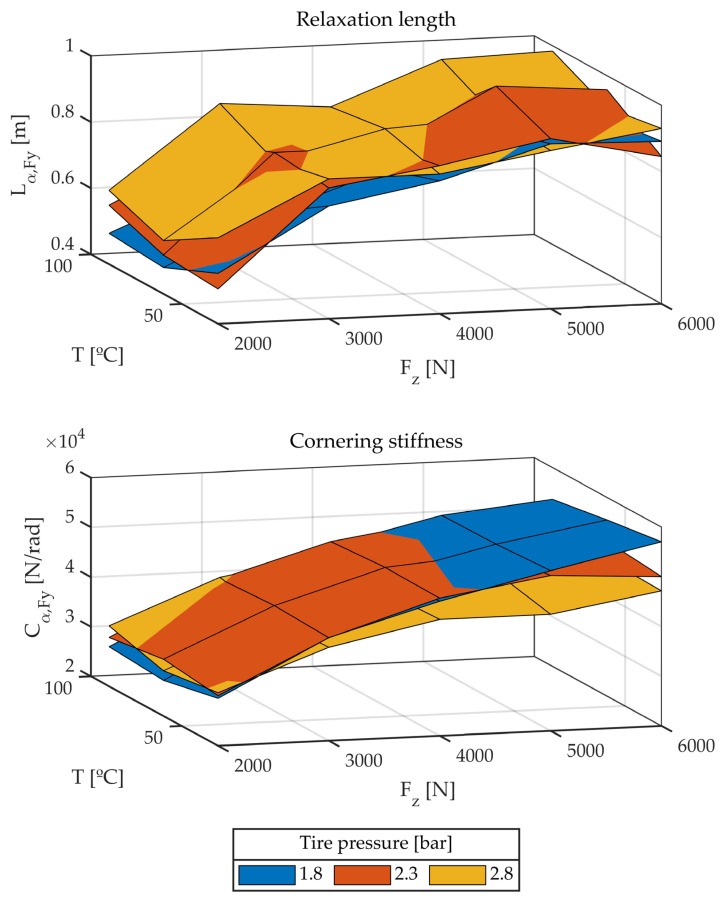
Relaxation length and cornering stiffness. Different temperatures, pressures, and vertical loads.

The test previously described is then performed, modifying three variables: pressure, temperature, and vertical load. Figure 12 shows the results of these tests.

Little predictable behavior can be observed: temperature seems to have practically no influence on any of the parameters studied, while pressure seems to have a slight effect, although no tendency can be identified. It could be said that all parameters increase slightly with pressure but, as shown in Figure 12, this is not always the case.

### 3.2. Non-Linear Tests

Non-linear tests are described in this section. These were performed outside the linear range of the tire, in this case, with a slip angle of −8 degrees.

**Figure 13:**
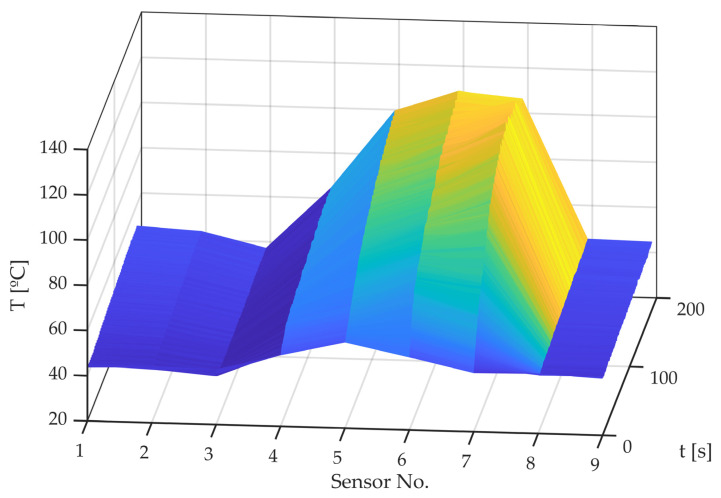
Measured temperatures for each sensor against time.

Figure 13 shows time evolution of each of the nine temperature sensor readings. It can be seen how the sidewall of the tire temperature (sensors 1, 2, 8, and 9) remains virtually constant over time. On the other hand, the tread working zone (sensors 5–7) heats up very quickly.

**Figure 14:**
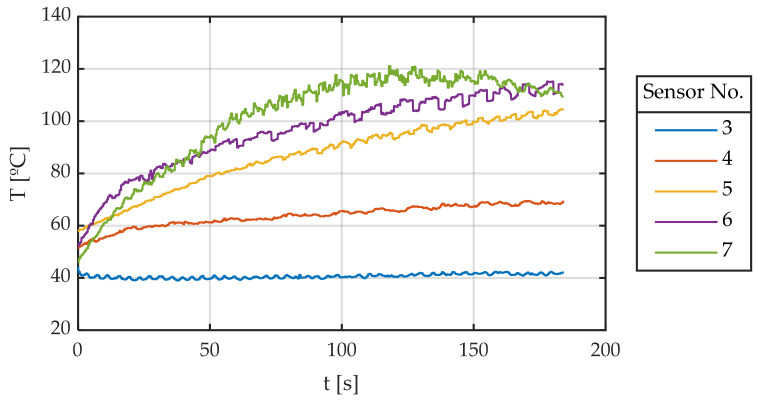
Tread temperatures against time.

**Figure 15:**
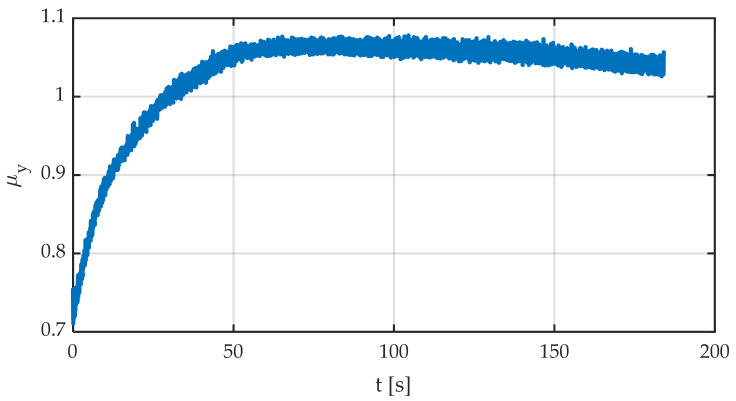
Friction coefficient against time.

Figure 14 shows the reading of the five sensors on the tire tread over time. The following can be observed:

- The tread area sensors that barely work (3 and 4) show how tire temperature slightly increases, reaching a stationary value relatively quickly.
- Sensors positioned in the tread area that contribute strongly to lateral force generation (sensors 5–7) show how the temperature in this area increases approximately linearly over time for sensors 5 and 6, while for sensor 7, it increases until it reaches a maximum around 120 °C and then it cools down slightly.
- If the temperature behavior of sensor 7 is analyzed, this maximum can be interpreted as follows. Since the friction coefficient increases with temperature up to a reference or optimum temperature and then decreases, this tire area generates less friction when this temperature is exceeded. As it generates less friction, it heats up less and cools down. It would be expected that, when it cools down, it would generate more friction and heat up again. What happens is that the area of the nearest sensors (5 and 6) has already exceeded 100 °C, so the total friction and heat generation decreases. As it can be seen in Figure 15, after approximately 100 s of testing, total friction decreases slightly. This moment occurs at the same time as the hardest working zones (5–7) exceed 100 °C. Consequently, the previously described Equation (3) is proposed to be used to provide a single value that represents the measurements of all the temperature sensors.

Figure 15 shows time evolution of the friction coefficient. This is a very illustrative graph since two main phenomena can be observed:

- The friction coefficient varies enormously with temperature. It starts at about a value of 0.7 and rises to a maximum of 1.05, representing an increase of 50%. This is one of the reasons for studying the influence of temperature on tire grip.
- The friction coefficient shows a maximum (not very sharp), then it decreases slowly.

**Figure 16:**
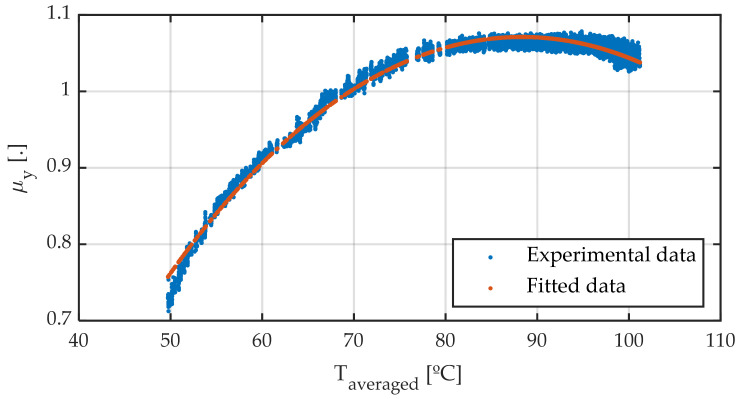
Friction coefficient against temperature. Different averaging methods.

As a novel contribution, this work includes determining an average temperature (Equation (3)), and a mathematical model that, using this averaged temperature, estimates the maximum friction coefficient. Figure 16 shows the friction coefficient versus the averaged temperature according to Equation (3). The fitting of data is carried out according to the newly proposed Equation (8):

(8) $$\mu_{y}\left(T\right)=\mu_{y}^{\max}+\left[1-\cosh\left(\frac{T_{\mathrm{averaged}}-T_{\mathrm{opt}}}{T_{\mathrm{dispersion}}}\right)\right]$$

where the fitted coefficients are shown in Table 4:

It is important to note that all parameters have a physical meaning: the maximum friction coefficient is given by $\mu_{y}^{\max}$ and occurs at temperature T_opt_. Additionally, T_dispersion_ is associated with the shape of the curve: a higher value indicates a flatter curve, whereas a lower one means a sharper one. It is observed that the adjustment is very accurate, allowing to estimate both the optimum working temperature and the value of maximum friction coefficient as a function of temperature.

This expression can be used to modify Pacejka’s magic formula, so that $\mu_{y}^{\max}$ is given by the MF, while the D term is obtained from the proposed Equation (8), resulting in (9):

(9) $$D_{y}=\left(\mu_{y}^{\max}+\left[1-\cosh\left(\frac{T_{\mathrm{averaged}}-T_{\mathrm{opt}}}{T_{\mathrm{dispersion}}}\right)\right]\right)\cdot F_{z}$$

## 4. Conclusions and Future Works

This work describes the importance of studying lateral dynamics between the tire and road. Experiments have been carried out on a test bench to determine the influence of temperature, longitudinal speed of the vehicle, steering frequency, vertical load, and tire pressure on lateral dynamics. Linear parameters associated with a first-order system have been studied: relaxation length and time constant. It has been shown how these parameters affect the maximum lateral friction coefficient outside the linear range. As contributions, the following are included:

- A new polynomial model is proposed to determine lateral relaxation length as a function of tire vertical load and longitudinal vehicle speed. It can be evaluated in real-time due to its low computational cost.
- A method is described to obtain a single representative temperature of the entire tread from an inhomogeneous distribution measured with infrared sensors. This method is based on Newton’s law of cooling, which is the fundamental heat dissipation mechanism of tires. In addition, the computational cost of determining this averaged temperature is relatively low.
- A new mathematical model to determine the maximum lateral friction coefficient as a function of representative temperature calculated according to the proposed method and its implementation into the MF set of equations.
- It has been indicated how some variables do not influence some of the parameters that characterize lateral dynamics so that they can be obviated for predictive control.
- Future work will include the following:
- Performing the same tests on different rolling surfaces: different belts on the tire test bench and especially real roads with an instrumented vehicle.
- Performing tests with different tires to determine the influence of other parameters, such as aspect ratio, tread width, etc.
- Carrying out longitudinal tests in order to be able to elaborate more complex combined models later.

## Figures and Tables

**Table 1:** Kistler RoaDyn P625 calibration certificate.

	F_y_	F_z_
Range [N]	±8000	±15,000
Linearity [% FS]	0.4	0.1
Hysteresis [% FS]	0.4	0.2
Sample rate [Hz]	5000	5000

**Table 2:** Values resulting from a least squares fitting of Equation (6).

Variable	Value	Units
c_1_	−1.4 × 10^−1^	m
c_2_	+2.1 × 10^−2^	s
c_3_	+1.9 × 10^−4^	m·N^−1^
c_4_	−1.6 × 10^−8^	m·N^−2^

**Table 3:** Values obtained by means of a least squares fitting of Equation (7).

Variable	Value	Units
d_1_	+5.2 × 10^4^	N/rad
d_2_	+2.7 × 10^0^	-
d_3_	+1.1 × 10^−4^	N^−1^

**Table 4:** Values obtained by means of a least squares fitting of Equation (8).

Variable	Value	Units
$\mu_{y}^{\max}$	+1.1 × 10^0^	-
T_dispersion_	+5.0 × 10^1^	°C
T_opt_	+8.8 × 10^1^	°C

## Data Availability

Not applicable.
